# Ensemble learning-based feature engineering to analyze maternal health during pregnancy and health risk prediction

**DOI:** 10.1371/journal.pone.0276525

**Published:** 2022-11-09

**Authors:** Ali Raza, Hafeez Ur Rehman Siddiqui, Kashif Munir, Mubarak Almutairi, Furqan Rustam, Imran Ashraf

**Affiliations:** 1 Department of Computer Science, Khwaja Fareed University of Engineering and Information Technology, Rahim Yar Khan, Pakistan; 2 Department of Information Technology, Khwaja Fareed University of Engineering and Information Technology, Rahim Yar Khan, Pakistan; 3 College of Computer Science and Engineering, University of Hafr Albatin, Albatin, Saudi Arabia; 4 Department of Software Engineering, School of Systems and Technology, University of Management and Technology Lahore, Lahore, Pakistan; 5 Information and Communication Engineering, Yeungnam University, Gyeongsan, Korea; Menoufia University, EGYPT

## Abstract

Maternal health is an important aspect of women’s health during pregnancy, childbirth, and the postpartum period. Specifically, during pregnancy, different health factors like age, blood disorders, heart rate, etc. can lead to pregnancy complications. Detecting such health factors can alleviate the risk of pregnancy-related complications. This study aims to develop an artificial neural network-based system for predicting maternal health risks using health data records. A novel deep neural network architecture, DT-BiLTCN is proposed that uses decision trees, a bidirectional long short-term memory network, and a temporal convolutional network. Experiments involve using a dataset of 1218 samples collected from maternal health care, hospitals, and community clinics using the IoT-based risk monitoring system. Class imbalance is resolved using the synthetic minority oversampling technique. DT-BiLTCN provides a feature set to obtain high accuracy results which in this case are provided by the support vector machine with a 98% accuracy. Maternal health exploratory data analysis reveals that the health conditions which are the strongest indications of health risk during pregnancy are diastolic and systolic blood pressure, heart rate, and age of pregnant women. Using the proposed model, timely prediction of health risks associated with pregnant women can be made thus mitigating the risk of health complications which helps to save lives.

## Introduction

Maternal health issues for women arise during pregnancy, childbirth, and the postnatal period [1]. At the time of pregnancy, women are at a higher risk of health complications which may lead to miscarriage and death in many cases. Every pregnancy stage must have a positive experience to ensure the good health of babies and women. Women who maintain their health during pregnancy and after childbirth, have a high probability of maintaining their health later in life. Such women have good birth outcomes, impacting childhood, adolescence, and adulthood of the children [2]. As a result, women’s health is important for both the mother and children. To reduce maternal and perinatal death and morbidity, actions across different stages are required. Before pregnancy, the overall lifestyle choices and health of women can affect maternal health risks. During pregnancy, high-quality care is necessary to ensure pregnancy health for mother and baby [3]. It is crucial to monitor both mother and baby’s health throughout the postpartum period [4], as both are at a higher risk of mortality during the first week of postpartum. The symptoms can be detected and managed early which can lower the risk of death.

The maternal mortality ratio refers to the number of women who die from pregnancy-associated factors per 100000 live births [5]. The world health organization (WHO) reports that approximately 300000 women died from pregnancy-associated causes in 2017 [6], which is 808 women every day. By analyzing the global maternal deaths per region, two-thirds (200000) of maternal deaths occur in sub-Saharan Africa and 19% (57000) of maternal deaths occur in South Asia alone [7]. In 2017, the five nations with the highest number of maternal fatalities are Tanzania (11000), Ethiopia (14000), the Democratic Republic of Congo (16000), India (35000), and Nigeria (67000) [6]. Pregnant women’s health factors such as age, and blood disorders including high or low blood pressure, blood glucose levels, body temperature, and heart rate can directly cause higher levels of complications during pregnancy resulting in loss of women’s pregnancy and death of both women and child. These health factors need to be controlled by special medications from the medical specialist whereby an early prediction of such health risks can potentially help the medical experts take appropriate actions to alleviate the probability of death.

In recent years, deep learning-based neural networks have been used in different domains to solve diverse problems [8]. Deep neural networks utilize layered architecture to learn the complex correlations among the variables. In deep learning, each successive layer utilizes the output from the previous layers. The deep learning techniques provide more accurate results, especially with the large amounts of input data [9]. Each successive layer is activated by receiving input from its neighboring neurons. Deep learning models are used for complex, precise, and accurate data analysis.

Deep learning-based models have been utilized for a rich variety of medical tasks like disease prediction using images, health records, and time-series data. Such models discover hidden patterns from medical data and help researchers and medical professionals to diagnose rapidly and accurately [10]. Deep learning provides the analysis of different infections accurately and helps doctors with better treatment. As a result, better medical decisions are made. Deep learning is also used for patient care and supporting clinicians [11]. Deep learning models analyze unstructured and structured medical records including diagnosis with better accuracy [12]. Medical applications [13] of deep neural networks include medical imaging, healthcare data analytics, maternal health care, drug discovery [14], genomics analysis [15], and many more. Taking due account of recent applications, suitability, and efficiency of deep learning models, this study adopts deep neural models to predict health risks associated with pregnancy and makes the following contributions

- A novel deep neural network architecture is proposed which considers decision trees, Bidirectional long short term memory (BiLSTM), and temporal convolutional network (TCN) to make DT-BiLTCN for feature extraction later to be used to train machine learning models. In this regard, support vector machine (SVM), extra tree classifier (ETC), logistic regression (LR), and decision tree classifier (DTC) are used to predict maternal health risk during women’s pregnancy. In addition, an ensemble model, BiLTCN comprising BiLSTM and TCN is also used.
- The maternal health exploratory data analysis (MHEDA) is performed to study the health conditions which serve as the strongest indicators to predict different maternal health risks during pregnancy.
- The performance of the DT-BiLTCN is further enhanced by two factors in essence including data resampling for data balancing and fine-tuning of hyperparameters of the proposed model. The synthetic minority oversampling technique (SMOTE) is utilized to balance the health dataset in this study while hyperparameters are tuned to select the set of best-fit hyperparameters for better performance.
- A comparative analysis of several existing models is carried out with the proposed approach to analyze its efficiency. The receiver operating characteristic (ROC) accuracy curve analysis is conducted to analyze the performance of the proposed BiLTCN model at different risk thresholds.

The rest of this study is organized as follows. Related work is discussed in Section 2. Section 3 describes the architecture and methodological details of the BiLTCN model. Results and discussions are given in Section 4. In the end, Section 5 provides the conclusion.

## Related work

The importance of maternal health led many researchers to devise models and approaches for the timely prediction of health risks during pregnancy using both traditional and machine learning techniques. Some studies focus on observing pregnant women’s conditions for analyzing and recording health risk factors while other aims at the prediction. For example, a pregnancy risk detection system (PRDS) is constructed to detect the pregnancy risk level based on the pregnant woman’s experienced symptoms [16]. The research observations are conducted continuously over time at Panimbang health center. Several health risk factors are observed like age over 35 or under 20 years old, having given birth before, history of disorder during pregnancy, and miscarriage. The pregnancy risk level is divided into three categories of high risk, low risk, and moderate risk.

Pregnant women’s health risk levels are predicted and monitored by using cloud-based machine learning techniques in Bangladesh [17]. The technique identified the maternal health risk intensity by analyzing the pregnant women’s health factors. A total of 1014 samples have been collected from numerous sources by utilizing wearable sensing devices. Python and WEKA toolkit platforms are used for model building. The maternal health risk levels are divided into low risk, mid risk, and high risk with the help of medical experts. The health factors like age, blood pressure, and blood sugar are identified as the key factors for high risk. Experimental results show that DT achieved a 97% accuracy score using hyperparameter tuning [18]. The pregnancy data is analyzed and utilized to build a machine learning classifier or health risk prediction during the pregnancy of women in [19]. The pregnancy data of 600 samples are collected from three medical centers in Bangalore. The employed DT model achieved a 71% accuracy score. The relative absolute error value is 99% and root related squared error value is 99% for the DT classifier. [20] predict health complications by using two machine learning-based classifiers. The study aims to reduce the fetal and maternal mortality rate by analyzing the pregnancy-related dataset. The classifiers are applied to identify the maternal health risk of pregnant women. Similarly, Naive Bayes (NB) and decision tree (DT) are employed in [21]. DT achieved an accuracy score of 66% while the relative absolute error value is 74% and root related squared error value is 110%.

Maternal mortality rate health risk factors are identified and observed using different data repositories in [22]. The study uses the root mean square error (RMSE) based on k-fold cross-validation to evaluate the proposed model performance. The world indicator 2015 (WDI-2015) dataset is utilized for the model building which contains 1350 samples from 1960 to 2015. The linear regression showed an RMSE of 0.709. The study asserts the need of reducing the rural population growth and ensuring necessities for better child care to reduce the pregnancy-related mortality rate. An efficient classification and regression tree (CART) binary DT approach is proposed to predict the high pregnancy risk using fetal health status in [23]. The cardiotocography dataset from UCI consisting of 2126 fetal cardiotocograms is utilized for risk prediction. Using a 5-fold cross-validation, it achieves an 88% accuracy.

Besides using the machine learning models on the collected data, some approaches devise telemedicine or electronic online monitoring systems for data collection. For example, a telemedicine framework is proposed to help health doctors in predicting pregnancy health risks in the Philippines [24]. The cloud-based dataset [25] of 97 samples was utilized to train and test four machine learning techniques such as DT, random forest (RF), k nearest neighbor (KNN), and support vector machine (SVM). RF achieved a 90% accuracy score by employing hyperparameter tuning. The telemedicine framework on imputing pregnant women data displayed the negative or positive cases concerning the possibility of pregnancy high-risk factors. Similarly, an Internet of things (IoT) based integrated system is developed in [26] to monitor the fetal and maternal signals for high-risk pregnancy predictions. IoT devices are used for fetal and maternal monitoring. A 1D convolutional neural network (CNN) [27] is utilized to predict the health risks during pregnancy. The maternal clinical factors and the heart rate of the fetal are monitored. The utilized maternal factors are diastolic/systolic blood pressure, oxygen saturation, temperature, heart rate, and uterine tonus activity. The proposed system achieved an accuracy score of 92% for fetal and maternal emergencies. Similarly, CNN is reported to have superior performance for distracted driver detection in [28].

Ensemble models show better performance than individual models, so they have been adopted for pregnancy health risk prediction as well. An ensemble classifiers-based [29] approach is utilized to predict the birth mode in this study [30]. The study provides the proper identification of health risk levels accomplice with pregnant woman delivery and helps reduce the mortality rate in Bangladesh. The research dataset consisting of 4493 samples is collected from the Demographic and Health Survey (BDHS-2014) in Bangladesh. The ensemble classifier achieved an 86% of accuracy score for birth mode classification. The maternal health risk prediction based on electronic health registries using an RF is proposed in [31]. The relevant features are selected by the recursive feature elimination (RFE) for the decision-making task. The systematic process of data analysis, preparation, and modeling are developed. The study uses several machine learning-based models with a grid search strategy using k-fold cross-validation and hyperparameters tuning. RF achieves an accuracy score of 93% in terms of maternal health risk prediction.

The discussion of the above-cited studies indicates that several aspects need further research efforts. First, the prediction accuracy is comparatively low for the majority of the works, except for a few single and ensemble models. Second, predominantly, studies focus on binary classification and do not consider the risk levels. Third, deep learning models are seldom studied. This study employs two deep learning models and predicts the health risks related to pregnant women into low, medium, and higher levels.

## Materials and methods

**Fig 1:**
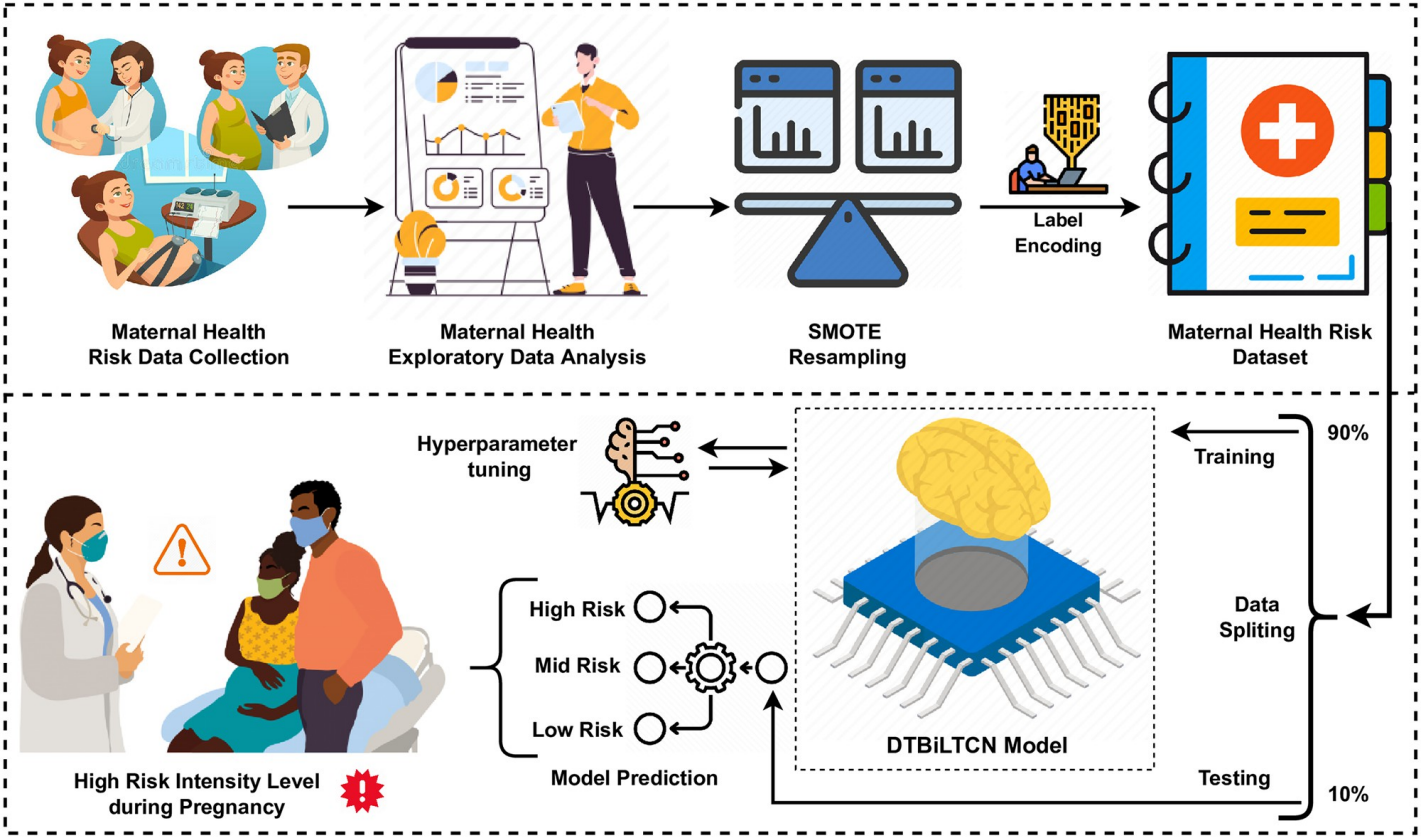
The methodological analysis of the proposed research study.

This section elaborates on steps related to employed techniques and methods to predict maternal risk. The flow diagram of the adopted methodology is visualized in Fig 1. It contains several steps which are followed sequentially.

- **Step 1:** The maternal health-related dataset is acquired in this step. The dataset is obtained from the Kaggle repository and is publicly available. Originally, Marzia et al. created the dataset using the IoT-based risk monitoring system [32]. The doctors structured the dataset and made it available for research purposes. The collected data contain various prominent health factors related to maternal health risk prediction.
- **Step 2:** MHEDA is applied to examine the pregnancy risk factors and get useful insights from the collected data. MHEDA contains numerous analysis graphs. The statistical data analysis, violin graphs, pie charts, feature relation analysis, and many more graphs are analyzed to get useful health factors and health conditions during pregnancy.
- **Step 3:** This step contains the dataset resampling technique implementation. The collected health dataset is imbalanced which hinders the full potential of the proposed approach. Training of the proposed model may become overfit due to class imbalanced data distributions. To balance the dataset, SOMTE is utilized.
- **Step 4:** The encoding of the category of health dataset is utilized in this step. The low risk, medium risk, and high-risk labels are converted to numeric forms 0, 1, and 2, respectively. The label encoding is applied to convert the target label into machine-readable labels. Now the dataset is in a structured format for the next research steps.
- **Step 5:** The dataset splitting is carried out in this step. The splitting depends upon the split of two portions of data one for proposed model training and the other portion of data for model testing and evaluations. The 80% of data is used for the train portion and 20% data for model evaluations.
- **Step 6:** The DT-BiLTCN deep learning-based model is built in this step. The DT-BiLTCN is a hybrid of DT, Bidirectional LTSM, and TCN models. It is tested and evaluated with 20% of the dataset. The DT-BiLTCN model is used for feature extraction. The extracted feature set is later used to train all the machine learning models and the evaluation is carried out using accuracy, classification report, F1 score, ROC curve accuracy, precision, and recall score.
- **Step 7:** Based on the model performance evaluation result, hyperparameter tuning is employed in this step. The model hyperparameter tunning is applied to get more accurate results from the proposed model. The number of epochs, model layers, vocabulary size, loss function, accuracy matrix, loss optimizer, and the total trainable parameters involved are the hyperparameters of the proposed model.

### Maternal health dataset

The maternal health data has been collected from different hospitals, community clinics, maternal health cares through the IoT-based risk monitoring system [33]. Marzia et al. from the Institution of Daffodil International University, Dhaka, Bangladesh created the dataset and it is publicly available [32]. The benchmark dataset is publicly available on Kaggle [34]. The dataset contains 7 features including age, SystolicBP, DiastolicBP, BS, BodyTemp, HeartRate, and RiskLevel as target classes. Table 1 describes the maternal health dataset using features-related information. The total maternal health data sample size is 1218.

**Table 1:** The maternal health dataset features related information.

Attribute	Non-null count	Data type	Description
Age	1218	Int64	Age in years when a woman is pregnant.
SystolicBP	1218	Int64	The upper value of Blood Pressure in mmHg is another significant attribute during pregnancy.
DiastolicBP	1218	Int64	The lower value of Blood Pressure in mmHg is another significant attribute during pregnancy.
BS	1218	Float64	Blood glucose levels are in terms of a molar concentration, mmol/L.
BodyTemp	1218	Float64	Pregnant women’s body temperature.
HeartRate	1218	Int64	A normal resting heart rate in beats per minute.
[RiskLevel	1218	Object	Predicted risk intensity level during pregnancy.

### Maternal health exploratory data analysis

MHEDA refers to the process of discovering dataset patterns, hypothesis tests, and assumption checks by utilizing the graphical data representations and statistics summary of the dataset. MHEDA helps to summarize the main dataset characteristics and features relation analysis. The feature relation analysis and data visualization methods help in the proposed model’s prediction process. Results of statistical dataset features analysis are given in Table 2. The feature statistic is based on the count, mean, standard deviation (std. dev.), minimum (min), 25%, 50%, 75%, and maximum (max) values. The analysis demonstrates that the dataset contains 1218 rows for each feature. The minimum values show the lowest limit, and the maximum values show the highest limit for all features. The minimum age of involved patients is 10, and the maximum age is 70 years. The analysis demonstrates that with the maximum age of 70 there is an indication of low risk during pregnancy. The systolic BP has a minimum value of 70, and the highest value is 160. Diastolic BP has a minimum value of 49, and the highest value is 100. Patients’ minimum and maximum heart rate values are 70 and 90. All the dataset features are analyzed statistically to check the behavior of each patient’s values in predicting the maternal health risk.

**Table 2:** The maternal health dataset features related information.

Feature	Count	Mean	Std. Dev.	Min	25%	50%	75%	Max
Age	1218.0	30.45	13.56	10.00	19.00	28.00	40.00	70.00
SystolicBP	1218.0	144.34	18.84	70.00	100.00	120.00	120.00	160.00
DiastolicDB	1218.0	77.36	14.07	49.00	65.00	80.00	90.00	100.00
BS	1218.0	9.05	3.51	6.00	6.90	7.50	9.43	19.00
BodyTemp	1218.0	98.69	1.39	98.00	98.00	98.00	98.00	103.00
HeartRate	1218.0	74.48	8.03	70.00	70.00	76.00	80.00	90.00

**Fig 2:**
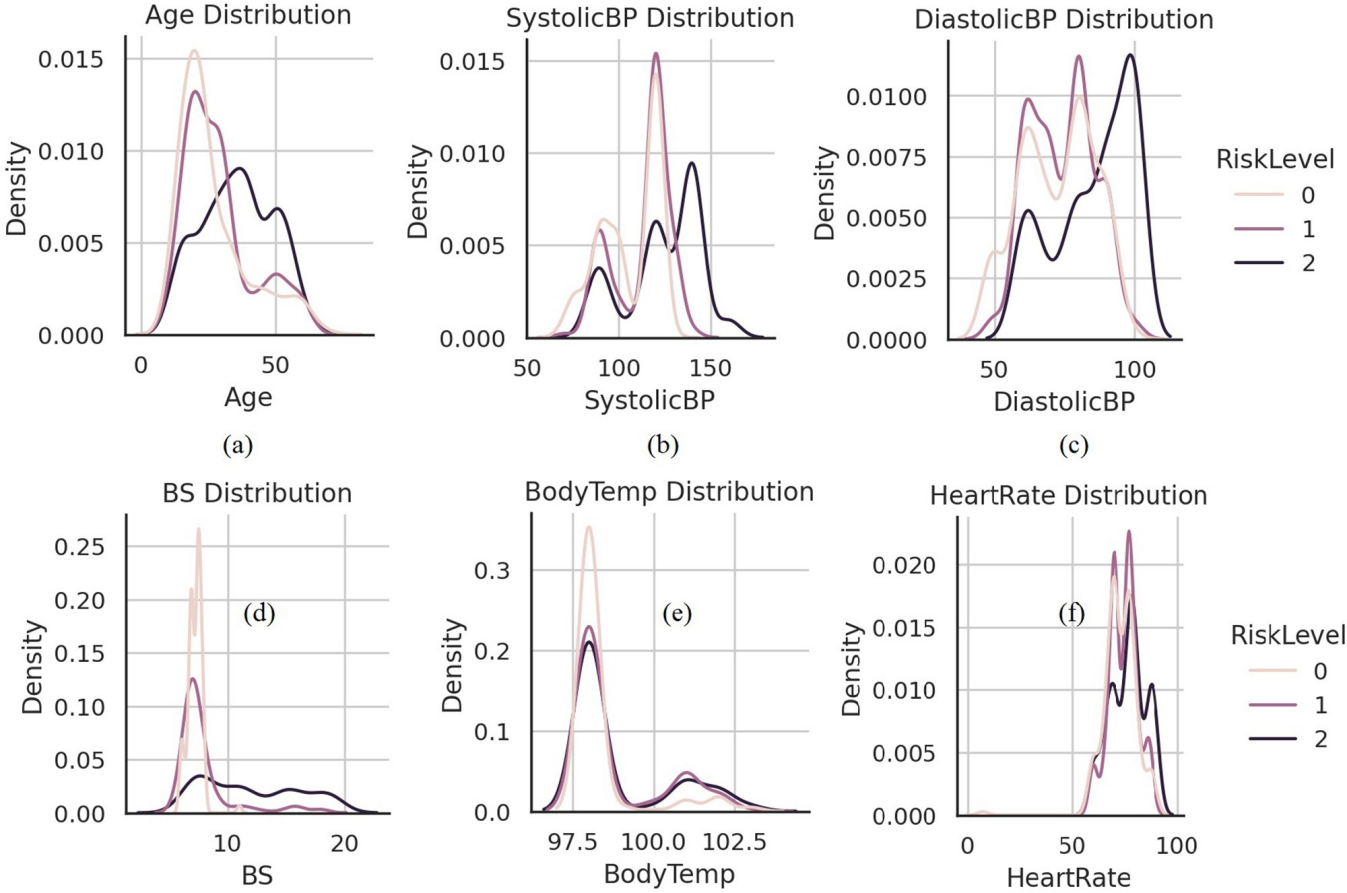
The dataset features distribution analysis concerning correlation to risk levels.

Fig 2 shows the analysis of different features of the collected dataset. It shows that each feature has a different correlation between high, medium, and low health risk levels indicating the importance of features to predict each class of risks. The bar chart distribution analysis between the age of pregnant women and the risk level is analyzed in Fig 2a. The analysis shows that from the age of 10 to 13 there is a high level of risk during pregnancy. In the age range of 18 to 30, the risk level is normal in all aspects. Between the age of 40 to 60 pregnant women face a high-risk level. The distribution analysis between the systolic blood pressure of pregnant women and the risk level is analyzed in Fig 2b. The analysis describes that systolic blood pressure of 140 causes a high-risk level. The 80 to 100 has a low-level risk of systolic blood pressure. The distribution analysis between the diastolic blood pressure of pregnant women and the risk level is analyzed in Fig 2c. The analysis shows that diastolic blood pressure at 100 causes high pregnancy risk levels.

**Fig 3:**
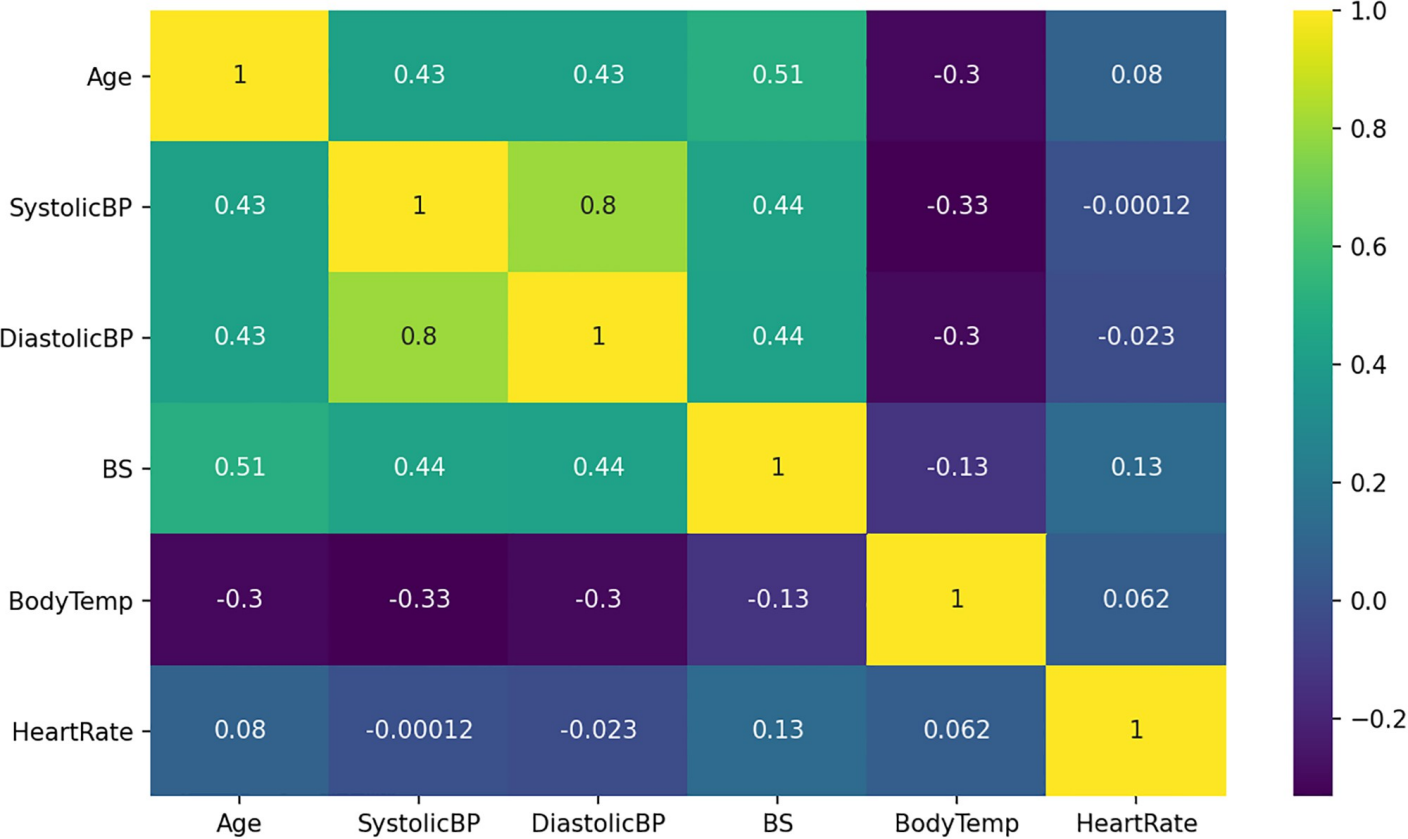
Statistical analysis of dataset features for correlation.

The correlation analysis is a statistical technique utilized to find out one feature’s change concerning the other feature of the dataset. We found the degree of the meaningful relationship and association among different dataset feature variables. The correlation analysis gives an association measure value between different dataset variables. The analysis demonstrates that the body temperature features are highly negatively correlated with all dataset features. The features systolic BP and diastolic BP have a low negative correlation with only the heart rate feature. Other dataset features have a positive correlation. A high positive correlation is between the systolic BP and diastolic BP features in context to the classification problem. The maternal health dataset feature correlation analysis is shown in Fig 3.

**Fig 4:**
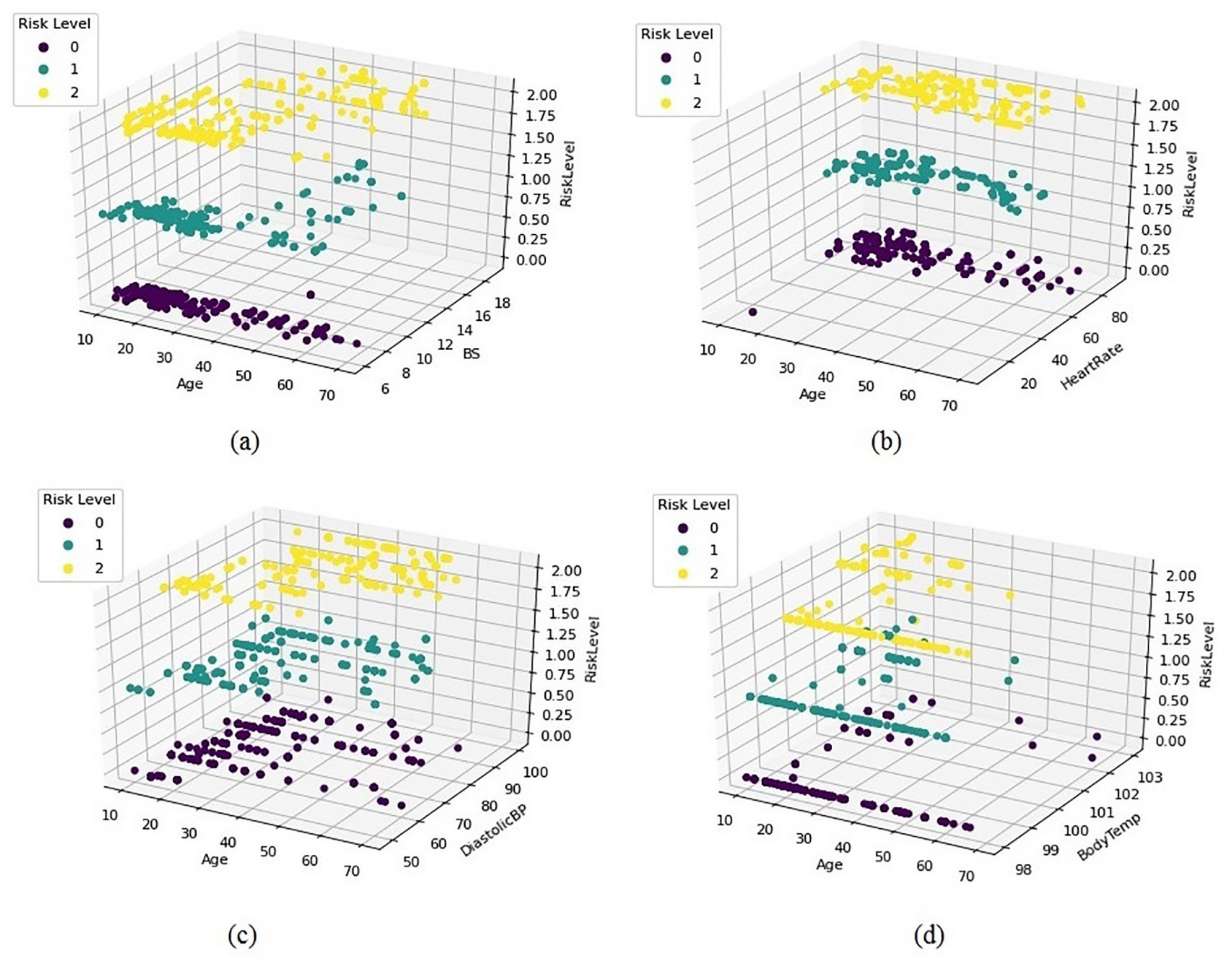
3D analysis of prominent features concerning maternal pregnancy risk level and age.

The 3-dimensional (3D) relation analysis of prominent dataset features with maternal pregnancy risk level and the age of the women are examined. The 3D scatter plots are used to plot data points on three axes to show the relationship between three variables. The conducted analysis is visualized in Fig 4.

The blood glucose (BS)levels in terms of the molar concentration, age, and risk level are analyzed in Fig 4a. We take the age, BS, and risk levels on the x, y, and z-axis, respectively to show the maternal health risk. It is observed that the risk level is low when the BS is between 6 to 8 and the age varies from 10 to 70. Risk level changes to medium when the BS is between 8 to 18 and age is between 10 to 50. The risk level is high when the BS is between 10 to 18 and the age is between 10 to 50.

In Fig 4b age, heart rate, and risk level is taken on the x, y, and z-axis, respectively. The graph shows that the risk level is low when the heart rate is above 60 and the age is from 10 to 70. There is a low-risk level when the age is 10 to 35. The risk level is medium when the heart rate is above 80 and the age is between 10 to 50. The risk level is high when the heart rate is above 80 and the age is between 10 to 70.

In Fig 4c age, diastolic blood pressure (BP), and risk level are taken on the x, y, and z-axis, respectively to show the maternal health risk. The risk level is low under the age of 70 and DBP is above 50. There are higher chances to remain at risk level low when the age is between 10 to 30 and DBP is above 60. When the age is under 50 and DBP is above 50 the risk level is medium. The risk level is high when the DBP is above 70 and the age is under 60.

In Fig 4d age, body temperature, and risk level are taken on the x, y, and z-axis, respectively. When the body temp is 97 and the age is between 10 to 70 the risk level is low. The graph also shows that when the age is under 30 the body temperature varies from 96 to 103 however the risk level remains low. When the body temperature is 97 and the age is under 40 then the risk level is medium and the graph also shows that the body temperatures vary from 97 to 103 under the age of 40 the risk level remains medium. The risk level is high when the body temperature is above 100 and under the age of 30.

**Fig 5:**
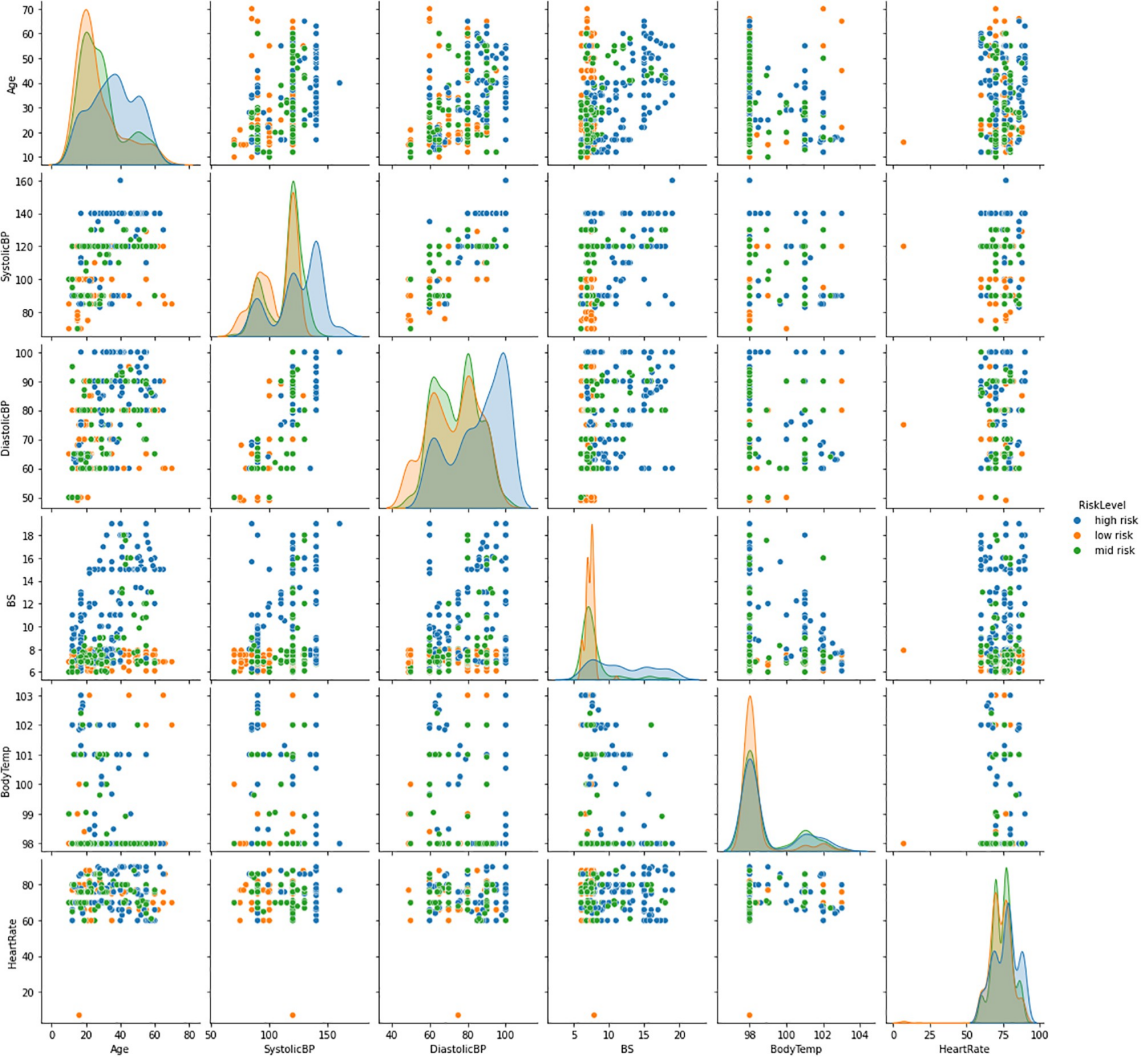
The pair plot analysis of dataset features.

The pair plot is utilized to find the best set of data features that elaborate a relationship between two features and is used to form the most separated clusters. The pair plot analysis among all the dataset features with the risk levels are visualized in Fig 5. This analysis demonstrated the distributions of every single feature with all other dataset features. The feature plots are in the matrix form in which the column represents the y-axis and the row represents the x-axis. For each feature, the main diagonal is the univariate distribution. In this analysis, the hue parameter is set to the value risk level feature. The feature scatters subplots are utilized to find the pairwise relationships among the distributions

### Maternal health data resampling

**Fig 6:**
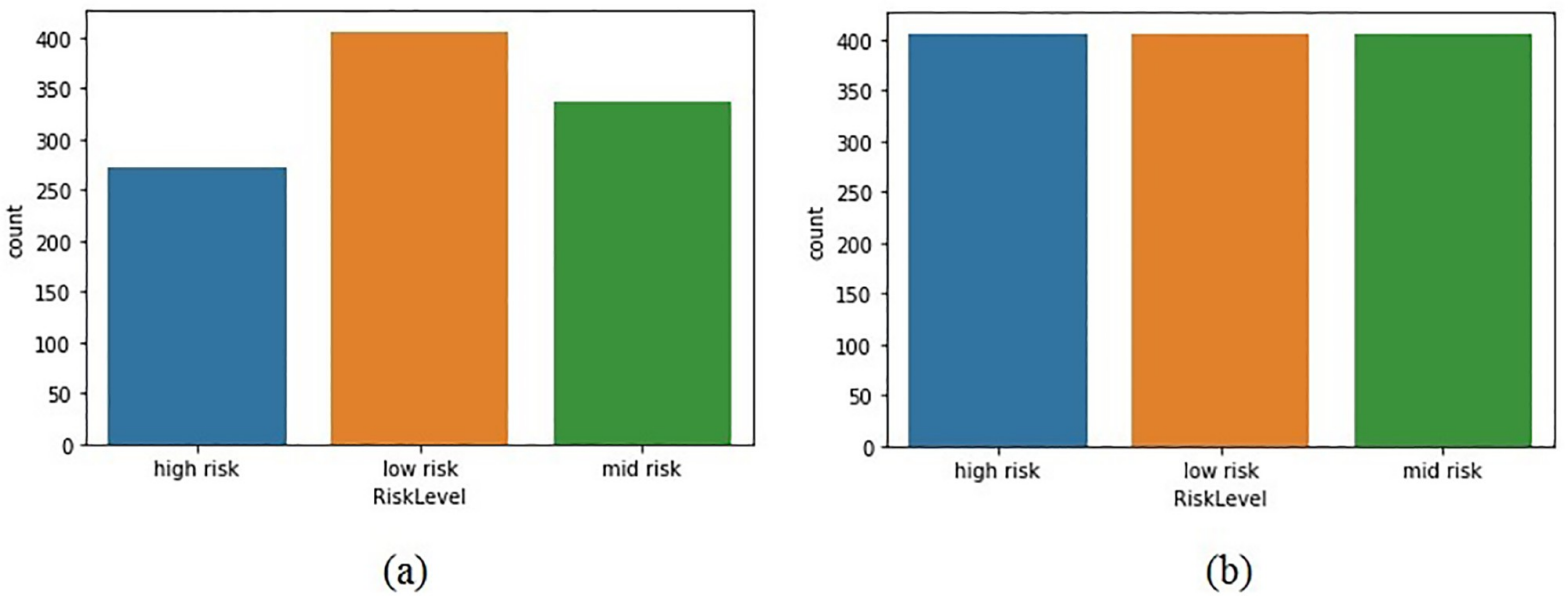
Number of samples for the dataset, (a) Before resampling, and (b) After applying SMOTE for resampling.

The maternal data resampling is conducted to achieve the best results from the proposed BiLTCN approach. Data resampling is applied to generate samples of equal weights and quantify the uncertainty of risk levels. SMOTE [35] is utilized to balance the maternal health dataset. From Fig 6, it can be observed that the number of samples for each class is balanced. The actual dataset contains 406 instances for low risk, 336 for mid risk, and 272 for high risk. By applying the data balancing technique, all classes have an equal 406 instances.

### Maternal risk label encoding

The label encoding is performed to convert the target category (label) into a machine (model) readable form [36]. The maternal risk Level categories low risk, mid risk, and high risk are encoded as 0,1,2 respectively. These encoded labels are used for maternal risk prediction by the BiLTCN model.

### Proposed DT-BiLTCN feature engineering

The proposed approach combines DT and BiLTCN for feature engineering on two rationales. First, DT and BiLTCN individually perform well for the task at hand. Owing to their performance, these models are combined in the proposed approach. In addition, we experimented with other combinations, however, the performance of those combinations was inferior. Secondly, it is empirically found that the use of prediction probabilities as features for model training yields better results as compared to using raw features. Therefore, this study proposes the use of prediction probabilities as the features for maternal health risk prediction.

**Fig 7:**
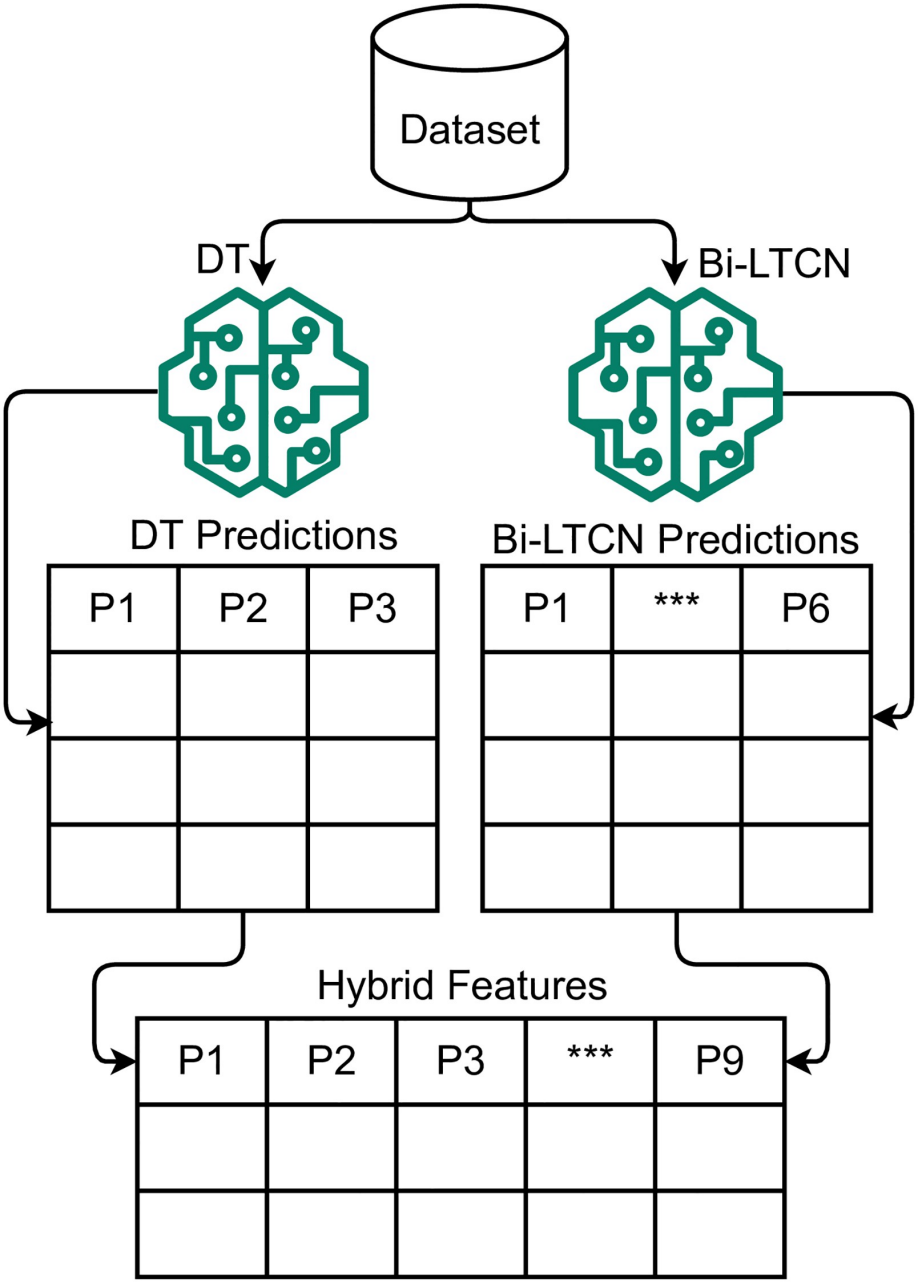
Graphical illustration of hybrid feature set using proposed DT-BiLTCN.

Machine learning and deep learning-based feature engineering approaches are deployed in this study to improve the performance of models. Feature engineering is performed using a combination of DT and BiLTCN. BiLTCN is the hybrid of Bidirectional LSTM [37] and TCN deep learning techniques. DT-BiLTCN approach is utilized as a feature engineering technique in this study. The feature set extraction and formation mechanism from the dataset using the DT-BiLTCN is shown in Fig 7.

**Fig 8:**
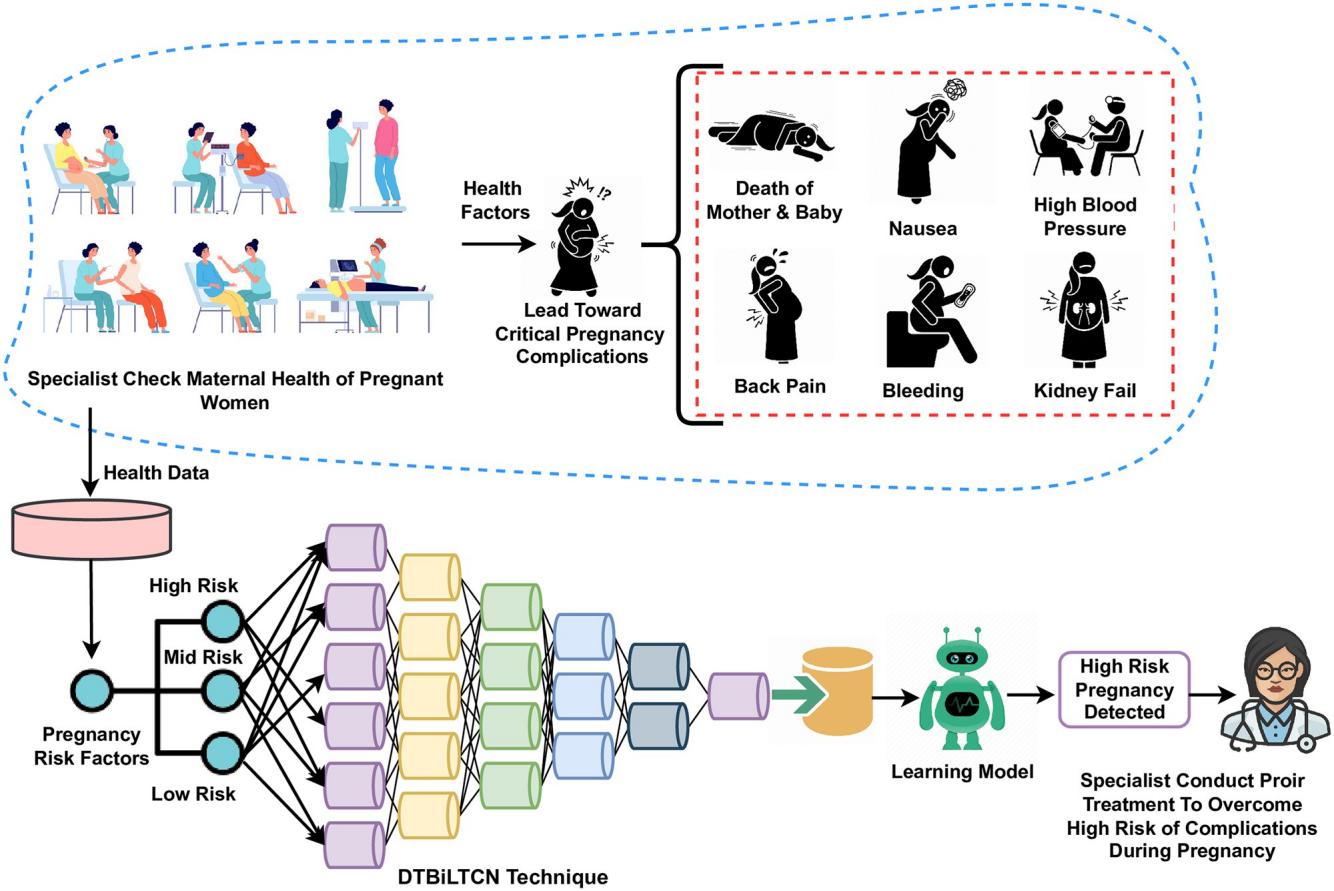
The maternal health risk analysis and BiLTCN model risk prediction.

The use of the DT-BiLTCN model for feature extraction, in the perspective of maternal health risk prediction, is illustrated in Fig 8. DT-BiLTCN approach is based on the hybrid of DT and BiLTCN models, so a brief description of each of these models is provided here.

#### Decision tree

DT is a supervised machine learning model and is commonly used for classification tasks. Classification or prediction is based on decision rules inferred from training data. DT has a tree-structured model, where the features of data are represented on internal nodes; the decision rules are represented by branches, and leaf nodes represent the outcomes. DT compares the values of the root attribute of the tree with the record attribute to predict the target class. The comparison of values continues until it reaches the leaf node of the tree. DT model is selected based on the reported performance in existing literature [38, 39]. Tree split criteria are important which determine the level of impurity and vary regarding classification and regression tasks. For example, Gini and Entropy are the commonly used criteria for classification while for regression tasks, mean squared error and half Poison deviance are used. This study uses Entropy with the following equation

$$\begin{array}{c} E\left(s\right)=\sum_{i=1}^{c}-p_{i}log_{2}p_{i} \end{array}$$ (1)

#### BiLTCN model

A deep learning-based novel BiLTCN model is also proposed in this study for performance comparison with the DT-BiLTCN model. The BiLTCN is the hybrid of Bidirectional LSTM and TCN deep learning networks. LSTM is an improved form of recurrent neural network (RNN) [40, 41] that allows information patterns to persist for a long time. There are three parts of LSTM which are referred to as gates. Forget gate is the first part, the input gate is the second part, and the output gate is the last part of LSTM they are defined as

$$\begin{array}{c} ForgetGate=f_{t}=\sigma \left(X_{t}*U_{f}+H_{\left(t-1\right)}*W_{f}\right) \end{array}$$ (2)

$$\begin{array}{c} InputGate=i_{t}=\sigma \left(X_{t}*U_{i}+H_{\left(t-1\right)}*W_{i}\right) \end{array}$$ (3)

$$\begin{array}{c} OutputGate=o_{t}=\sigma \left(X_{t}*U_{o}+H_{\left(t-1\right)}*W_{o}\right) \end{array}$$ (4)

where *X*_*t*_ shows the input for the current timestamp, *U*_*f*_ is the weight attached with the input, (*t* − 1) is the hidden state for the previous timestamp, *W*_*f*_ is the weight matrix attached with the hidden state, *U*_*i*_ is the input weight matrix, *H*_(*t* − 1)_ is the hidden state of the previous timestamp, and *W*_*i*_ is the weight matrix of input attached with a hidden state.

A BiLSTM [42] model contains two LSTM models where one LSTM takes the input information sequence in the forward direction (past to future) and the other LSTM in the backward direction (future to past). The BiLSTM input flow in both directions is used to preserve the future and past sequence information. The input *X* information sequence is computed in the forward and backward direction shown in Eqs 6, and 7. The final output cell *Y* at time *t* is constituted in both directions as in Eq 8.

$$\begin{array}{c} X=(X_{1},X_{2},...,X_{n}) \end{array}$$ (5)

$$\begin{array}{c} {\vec{h}}_{t}=\left({\vec{h}}_{1},{\vec{h}}_{2},...,{\vec{h}}_{n}\right) \end{array}$$ (6)

$$\begin{array}{c} {\overset{\leftarrow }{h}}_{t}=\left({\overset{\leftarrow }{h}}_{1},{\overset{\leftarrow }{h}}_{2},...,{\overset{\leftarrow }{h}}_{n}\right) \end{array}$$ (7)

$$\begin{array}{c} {\hat{y}}^{<t>}=g\left(W_{y}[{\vec{h}}_{t},{\overset{\leftarrow }{h}}_{t}]+b_{y}\right) \end{array}$$ (8)

The TCN [43] model is a variation of the CNN and is commonly used for sequence modeling tasks. The TCN working is to encode information from an input sequence. The TCN model has longer memory as compared to RNN with equal capacity. TCN utilizes causal convolutions, where at time *t* an output is convolved with only elements from the time *t* and the prior from the previous layer [44]. TCN utilizes a fully convolutional network (FCN) architecture [45]. The input layer has the length of each hidden layer. In the causal convolutional layer zero padding is added to the length (kernel size—1) to keep consequent layers the same length as the previous layer.

$$\begin{array}{c} TCN=1DFCN+CausalConvolutions \end{array}$$ (9)

$$\begin{array}{c} F\left(x_{t}\right)=\left(x*f\right)\left(t\right)=\sum_{j=0}^{k-1}f_{t}^{j}x_{t-j}\;x_{\leq 0}=0 \end{array}$$ (10)

The dataset samples are fed into the DT and BiLTCN models for training which provide the predicted class probabilities as the output. However, instead of using class probabilities for prediction, they are used to train machine learning models. The feature set obtained from DT and BiLTCN are combined to make the hybrid feature set and is represented by *V*_*hf*_

$$\begin{array}{c} V_{hf}=\left\{DT_{f},BiLTCN_{f}\right\} \end{array}$$ (11)

where *DT*_*f*_ and *BiLTCN*_*f*_ are the feature sets provided by the DT and BiLTCN, respectively, and are calculated as

$$\begin{array}{c} \left\{P{\left(c_{1}\right)}_{DT},P{\left(c_{2}\right)}_{DT},...,P{\left(c_{n}\right)}_{DT}\right\}=DT\left(X\right) \end{array}$$ (12)

$$\begin{array}{c} \left\{P{\left(c_{1}\right)}_{Bi},P{\left(c_{2}\right)}_{Bi},...,P{\left(c_{n}\right)}_{Bi}\right\}=BiLTCN\left(X\right) \end{array}$$ (13)

where *P*(*c*_1_)_*DT*_ and *P*(*c*_1_)_*Bi*_ show the probability of class 1 from the DT and BiLTCN models, respectively when the input data *X* is fed into these models. The *X* is the input dataset features (independent variables) that contain the whole records.

The proposed BiLTCN model build configuration parameters are analyzed in Table 3. The table contains an analysis of the number of layers, the neuron units, layer activation function, output shape of layer, and the total parameters used. The first layer of the model is the embedding layer [46] which contains 50000 neuron units, (None, 6, 64) output shape, and 3200000 total parameters used. The second layer is the BiLSTM layer having 128 neuron units, (None, 6, 256) output shape, and 197632 total parameters are used. The next layer is the TCN layer having 64 neuron units, (None, 64) output shape, and 201536 total parameters are used. The next layers are the family of dense layer networks. The 3 dense layers have 32, 16, 8 neuron units, (None, 32), (None, 16), (None, 8) output shape, ReLU is activation function, and 2080, 528, 136 total parameters are used, respectively. The last layer is the output layer having 3 neurons output units with Softmax as activation function, (None, 3) output shape, and 27 are the total parameters.

**Table 3:** The proposed BiLTCN model configuration parameters.

Layers	Unit	Activation Function	Output shape	Parameters
The Embedding layers.	50000	N/A	(None, 6, 64)	3200000
The Bidirectional LSTM layers.	128	N/A	(None, 6, 256)	197632
The Temporal Convolutional Network layers.	64	N/A	(None, 64)	201536
The Dense layers.	32	ReLU	(None, 32)	2080
The Dense layers.	16	ReLU	(None, 16)	528
The Dense layers.	8	ReLU	(None, 8)	136
The Dense layers.	3	SOFTMAX	(None, 3)	27

**Fig 9:**
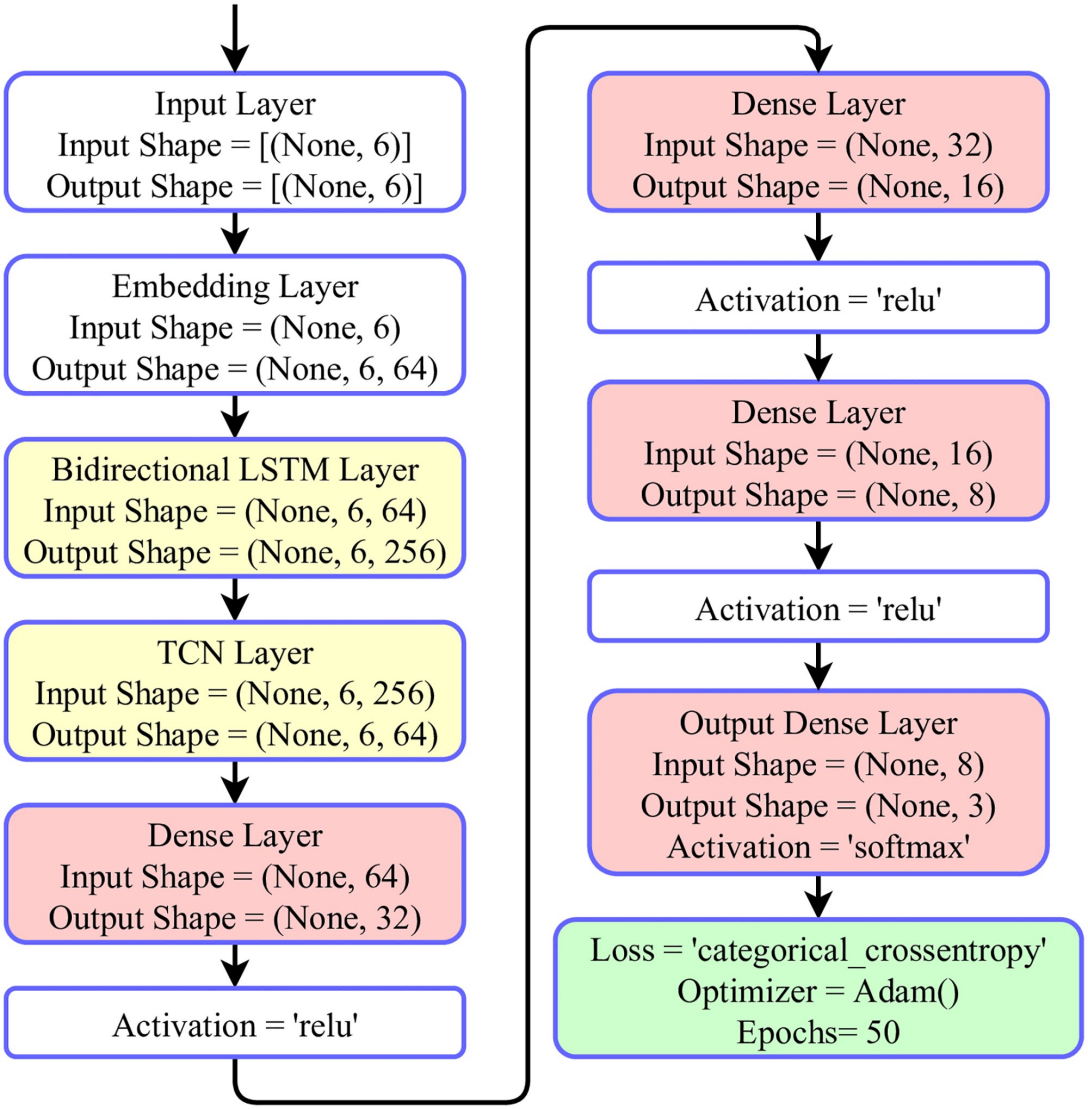
The architectural layers analysis of the BiLTCN approach.

The layered architectural analysis [47] of our proposed approach is illustrated in Fig 9. The first layer of the architecture is the input layer having the (None, 6) input and output shape. The second layer is the Embedding layer having the (None, 6) input shape and (None, 6, 64) output shape. Then in the layer stack, the Bidirectional LSTM layers are involved. The Bidirectional LSTM layer has an input shape of (None, 6, 64) and an output shape is (None, 6, 256). The next layer in the stack is the Temporal Convolutional Network layers that have an input shape of (None, 6, 256) and output shape of (None, 64). Then a family of 4 dense layers is involved. The last layer is the output layer of the architecture. The output layer has the input shape of (None, 8) and the output shape of (None, 3). This analysis demonstrated the layered stack of the proposed BiLTCN approach.

The hyperparameter tuning is applied to find out a set of best-fit hyperparameters [48] for the proposed BiLTCN model. The involved hyperparameter is the number of epochs, number of layers, model vocabulary size, the loss function, accuracy matrix, loss optimizer, and the total trainable parameters. The final best-fit hyperparameters for the BiLTCN model are given in Table 4. The proposed model is trained for 50 epochs. The model contains seven layers and the vocabulary size is 50000. The categorical cross-entropy is used as the loss function. The loss optimizer utilized is Adam with a learning rate of 0.001. The total number of model parameters is 3601939.

**Table 4:** The proposed model compilation parameters with hyperparameter tuning.

**Proposed Model**	**Hyperparameters**						
	Epochs	Layers	Vocabulary size	Loss function	Matrix	Optimizer	Total Parameters
BiLTCN	50	7	50000	Categorical cross-entropy	Accuracy	Adam (learning rate = 0.001)	3,601,939

## Results and discussions

This section contains the experimental results of machine learning models with respect to different perspectives. The performance of models is evaluated using imbalanced and balanced datasets. BiLTCN and DT are both evaluated as feature extractors, as well as classifiers. The performance of the proposed DT-BiLTCN is compared with BiLTCN as well regarding the feature extractor.

### Experiment setup

All experiments are performed using HP EliteBook 8440p with CPU 2.53GHz 2 cores, Intel core i5, having a RAM of 4.00 GB. The Python TensorFlow tool [49] is utilized for proposed model building and performance evaluations. Data splitting helps to optimize hyperparameters of models, estimate the model generalization performance, and avoid model overfitting [50]. We have split the maternal health data into 0.8 for training purposes and 0.20 for testing purposes. The random state instance value is 42 for data spiting. The utilized evaluation metrics are recall, precision, accuracy, F1 score, and ROC curve accuracy score values.

### Performance metrics

The accuracy shows the total number of accurate predictions from the predictive model out of all the predictions. It is calculated as

$$\begin{array}{c} Accuracy=\frac{TPR+TNR}{TPR+FPR+TNR+FNR} \end{array}$$ (14)

where TPR is the true positive rate and shows correctly identified by our predictive model, FPR is the false positive rate and incorrectly identified positive samples, TNR is the true negative rate showing the correctly predicted negative samples and FNR is the false negative rate and indicates the incorrectly identified negative samples.

Precision is the proportion of accurately predicted samples by the proposed model to all expected positive data samples. It is calculated using

$$\begin{array}{c} Percision=\frac{TPR}{TPR+FPR} \end{array}$$ (15)

Recall shows the preciseness of the model and is calculated using

$$\begin{array}{c} Recall=\frac{TPR}{TPR+FNR} \end{array}$$ (16)

The F1 sore performance metric is based on the combination of recall and precision and is calculated by taking the harmonic mean of recall and precision.

$$\begin{array}{c} F1-score=\frac{2*Precision*Recall}{Precision+Recall} \end{array}$$ (17)

The ROC curve accuracy performance metric is the probability curve that shows the TPR in comparison to FPR at different threshold values. The higher values of the ROC, the superior the proposed learning technique classification performance.

$$\begin{array}{c} ROC\;accuracy=\int_{0}^{1}TPR\left(FPR\right)dFPR \end{array}$$ (18)

### Results of machine learning models using original features

Initial experiments are carried out using the imbalanced data with different train and test split ratios to analyze the performance of deployed models. SVM, ETC, LR, and DTC are employed for these experiments, and results are provided in Table 5. Among the employed models, the DTC achieves the highest accuracy of 80 each for 80:20 and 85:15 train-test split ratios. Due to an imbalanced dataset, predominantly the models perform poorly for maternal health risk prediction.

**Table 5:** Comparative analysis of accuracy score of employed approaches based on original dataset features with imbalanced data.

Data split (Train:Test)	SVM	ETC	LR	DTC	BiLTCN
90:10	66	73	65	76	76
80:20	60	67	56	80	78
85:15	60	67	56	80	75
70:30	60	70	60	73	79

Further experiments are performed using the SMOTE balanced dataset for making equal samples for three classes of the dataset and results are displayed in Table 6. Indicating an accuracy of 84%, BiLTCN outperforms other models when used with the balanced dataset. It is a high individual accuracy as compared to models’ performance with the imbalanced dataset, so for further experiments, only the balanced dataset is used.

**Table 6:** Comparative analysis of the accuracy of employed approaches based on original dataset features using SMOTE-balanced data.

Data split (Train:Test)	SVM	ETC	LR	DTC	BiLTCN
90:10	57	61	50	79	81
80:20	58	70	85	78	73
85:15	57	71	56	80	84
70:30	58	70	56	78	77

#### Performance evaluation of BiLTCN

Experiments are performed to analyze the accuracy and other performance metrics for the proposed BiLTCN model. Epoch-wise results regarding the training accuracy and loss are presented in Table 7 indicating an average accuracy of 0.88 for the BiLTCN.

**Table 7:** Training accuracy and loss of BiLTCN concerning the number of iterations.

Epoch	Accuracy	Loss	Time	Epoch	Accuracy	Loss	Time
1	0.8819	0.2259	7s 219ms	26	0.8881	0.2175	6s 201ms
3	0.8819	0.2289	6s 198ms	28	0.8850	0.2172	6s 202ms
5	0.8901	0.2220	6s 200ms	30	0.8871	0.2172	6s 203ms
7	0.8922	0.2250	6s 202ms	32	0.8912	0.2166	6s 208ms
9	0.8912	0.2221	6s 201ms	34	0.8871	0.2130	6s 203ms
11	0.8891	0.2202	6s 202ms	36	0.8819	0.2189	6s 202ms
13	0.8901	0.2266	6s 205ms	38	0.8912	0.2174	6s 201ms
15	0.8871	0.2261	6s 201ms	40	0.8860	0.2131	6s 202ms
17	0.8891	0.2242	6s 206ms	42	0.8871	0.2144	6s 205ms
19	0.8645	0.2997	6s 203ms	44	0.8819	0.2133	6s 202ms
21	0.8840	0.2373	6s 201ms	46	0.8881	0.2102	6s 204ms
23	0.8891	0.2338	6s 203ms	48	0.8881	0.2124	6s 203ms
25	0.8881	0.2326	6s 202ms	50	0.8891	0.2184	6s 203ms

Experimental results in Table 8 demonstrate the classification report for each maternal health risk category. The low-risk level category (0) has an 80% precision score, 82% recall score, and 81% F1 score. The medium risk level category (1) has an 80% precision score, 76% recall score, and 78% F1 score. The high-risk level category (2) has a 90% precision score, 89% recall score, and 90% F1 score. Averaged scores for precision, recall, and F1 are also provided. The precision score for micro, macro, and weighted average is 83% each while the samples-average score is 82%. Averaged scores for recall and F1 also correspond to the precision scores and show better results using the balanced dataset.

**Table 8:** Results for the BiLTCN for all classes.

Class	Precision%	Recall%	F1 score%
Low risk	80	82	81
Mid risk	80	76	78
High risk	90	89	90
Micro-average	83	82	82
Macro-average	83	82	83
Weighted-average	83	82	82
Samples-average	82	82	82

The BiLTCN approach evaluation results with hyperparameter tuning are demonstrated in Table 9. The 50 epochs are used for model training. The training accuracy achieved by the model is 88% while the testing accuracy, precision, recall, and F1 scores are 84% each. BiLTCN obtains a ROC accuracy (micro) score of 97% for maternal health risk prediction.

**Table 9:** BiLTCN model results with hyperparameter tuning.

Epochs	Accuracy (%)		Precision%	Recall%	F1%	ROC accuracy(micro)%
	Training	Testing				
50	89	82	83	82	82	95

**Fig 10:**
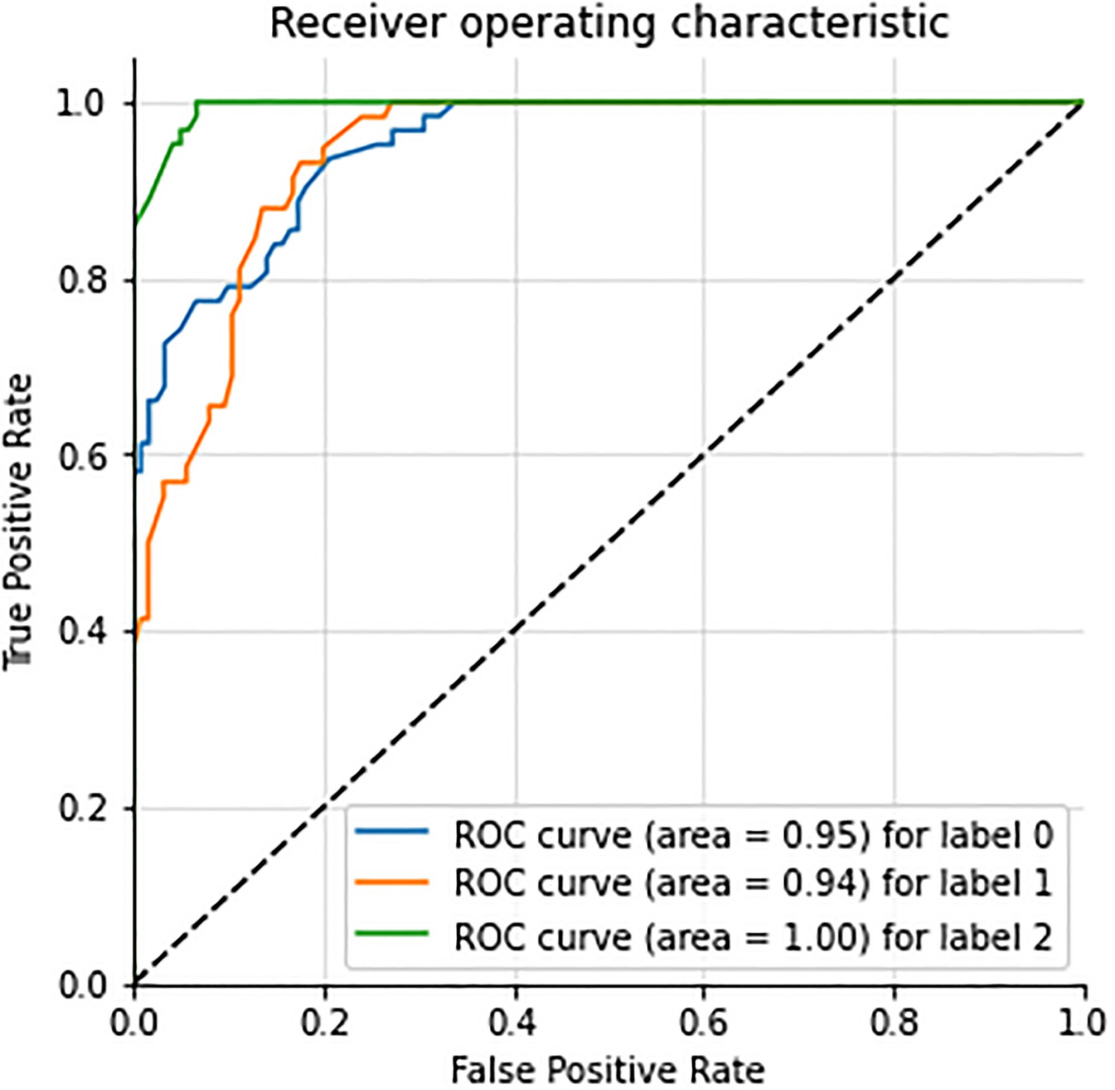
ROC curve accuracy analysis of BiLTCN.

The ROC accuracy curve analysis of the BiLTCN approach is examined in Fig 10. It shows that the low-risk class achieves a 95% ROC accuracy, the mid-risk class achieves a 94% ROC accuracy, and the ROC accuracy for the high-risk class is 99%. These results show that the BiLTCN approach is better than other employed machine learning models for predicting maternal health risks.

**Fig 11:**
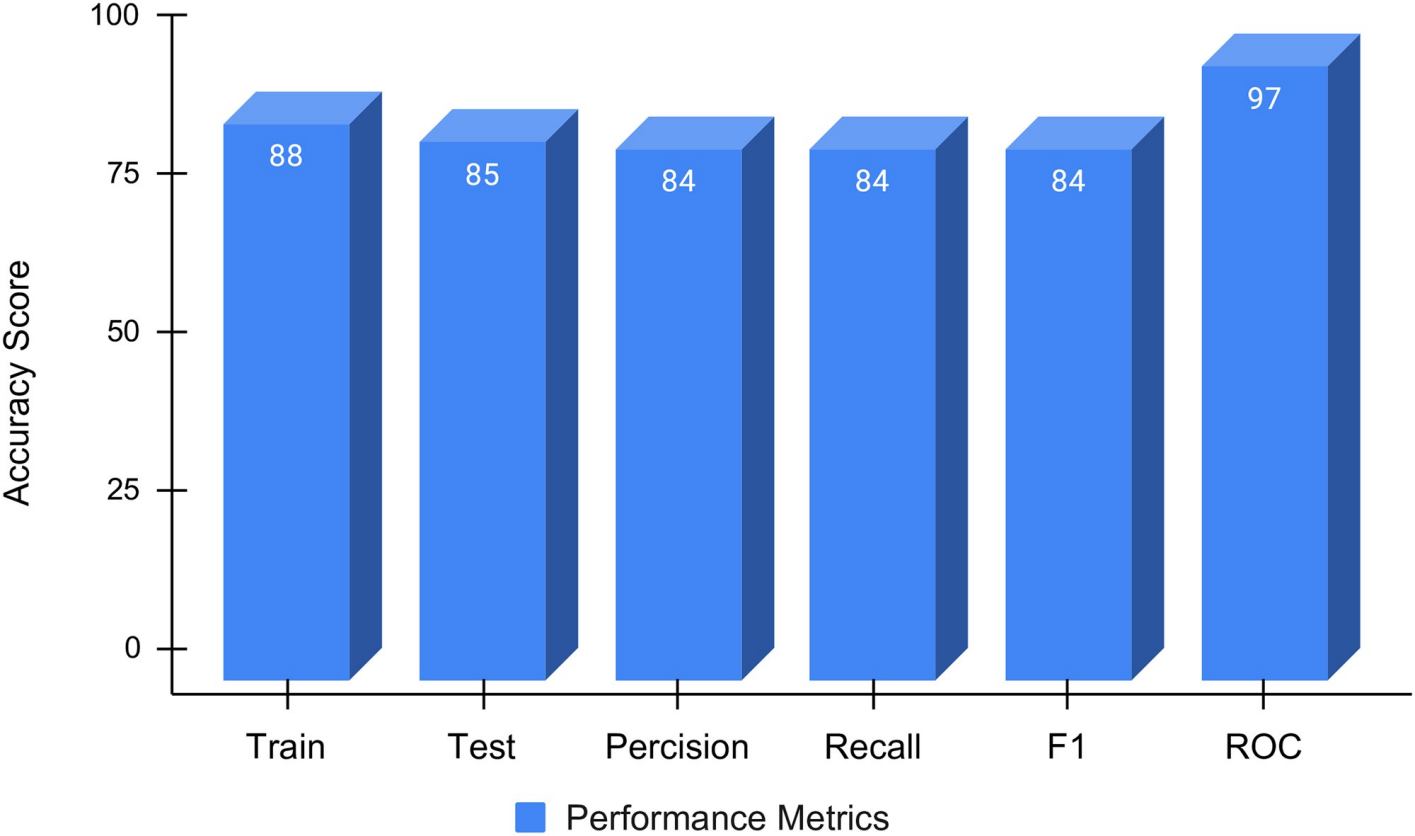
BiLTCN approach performance analysis.

The results of the BiLTCN are summarized in Fig 11 regarding the average recall, precision, F1 score, and ROC. The higher the ROC accuracy score, the better the classification model. The micro-ROC accuracy score of the employed model is 97% which demonstrates the best performance score.

### Performance analysis using proposed DT-BiLTCN feature engineering

The performance analysis with the proposed DT-BiLTCN technique is carried out in this section. Experiments are performed using both the imbalanced and balanced datasets. Table 10 shows the results with the imbalanced dataset. Results indicate that SVM shows superb performance as compared to other models even with the imbalanced dataset using the proposed approach and obtains a 95% accuracy.

**Table 10:** Averaged results of models using the proposed DT-BiLTCN feature extraction technique with the imbalanced dataset.

Model	Accuracy%	Precision%	Recall%	F1 score%
DTC	90	91	90	90
LR	92	93	92	92
ETC	90	91	90	90
KNN	89	91	88	89
RFC	90	91	90	90
**SVM**	**95**	**95**	**96**	**95**

The feature set is extracted by using DT-BiLTCN which is input to machine learning models for maternal risk prediction. Table 11 shows the comparative analysis of the accuracy score among the applied learning techniques. Results show that using the DT-BiLTCN-based features, the performance of the models is substantially improved. The highest accuracy is obtained by the SVM which is 98%. Similarly, its precision, recall, and F1 scores are also 98% each which shows superior performance as compared to other models.

**Table 11:** Averaged results of applied learning models using the proposed DT-BiLTCN feature extraction technique.

Model	Accuracy%	Precision%	Recall%	F1 score%
DTC	93	94	93	93
LR	97	97	97	97
ETC	93	94	93	93
KNN	95	96	95	95
RFC	95	95	95	95
**SVM**	**98**	**98**	**98**	**98**

The category-wise classification report of the SVM is given in Table 12. Results show that SVM achieves a 100% precision for the low-risk class, while 98% and 97% for medium-risk and high-risk categories of maternal health risks. Recall and F1 scores also correspond to high performance for all categories of maternal health risk which indicates the superiority of the proposed DT-BiLTCN feature engineering approach.

**Table 12:** SVM category-wise classification report.

Class	Precision%	Recall%	F1 score%
Low risk	100	95	98
Mid risk	98	100	99
High risk	97	100	99
**Average**	**98**	**98**	**98**

In addition to using the proposed DT-BiLTCN approach, DT and BiLTCN are also used as feature engineering approaches to carry out the performance comparison and the results are provided in Table 13. Results suggest that models perform superbly better when trained on the features extracted using the DT model. The class probabilities used by the DT are used to train the model, which improves their learning process and elevates their maternal health risk prediction performance. Results using DT features are far better than using the BiLTCN features.

**Table 13:** Comparative analysis of DT and BiLTCN as feature extraction techniques.

Model	Accuracy%		Precision%		Recall%		F1 score%	
	BiLTCN	DT	BiLTCN	DT	BiLTCN	DT	BiLTCN	DT
DTC	80	95	80	95	80	95	80	95
LR	38	95	32	95	40	95	31	95
ETC	81	95	81	95	81	95	81	95
KNN	68	93	69	93	68	94	68	93
RFC	79	95	79	95	78	95	78	95
SVM	50	95	49	95	49	95	49	95

**Fig 12:**
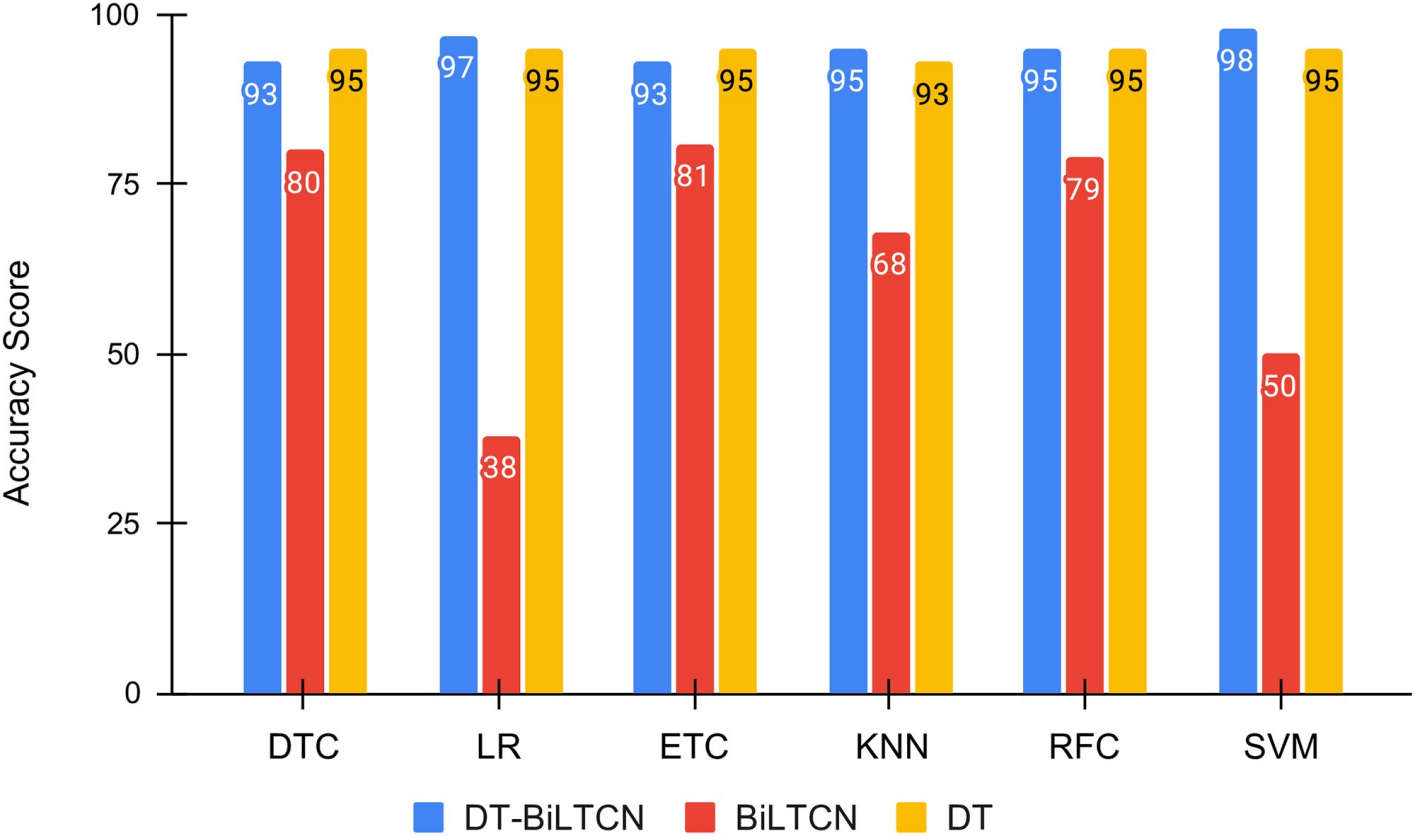
Comparative analysis of proposed DT-BiLTCN feature extraction with DT and BiLTCN.

Despite the better performance of machine learning models with DT features, the performance of DT-BiLTCN features is still better, as shown in Fig 12. Results show that the highest accuracy of 98% is achieved when DT-BiLTCN features are used. Since the proposed approach combines features from better-performing DT and BiLTCN models, it contains an appropriate feature set that improves the training of the models and elevates their performance.

### Validation of proposed DT-BiLTCN

The proposed approach is validated using 10-fold cross-validation using the superior performing SVM model which obtained a 98% accuracy score. In addition, the results of all employed models are also provided in Table 14. As per the given results, the superior performance of SVM is validated where it obtains a 94% accuracy with a standard deviation of only ±0.02.

**Table 14:** The 10-fold cross-validation analysis of employed approaches with proposed DT-BiLTCN technique.

Model	Accuracy%	Standard deviation
DTC	92	±0.05
LR	93	±0.02
ETC	87	±0.02
KNN	90	±0.02
RFC	91	±0.02
**SVM**	**94**	**±0.02**

### Comparison of probability-based and original features

The original maternal health dataset features are small in size and are not linearly separable to a great extent. This non-linearly separable behavior results in poor performance of machine learning models. The proposed feature engineering technique is utilized to design the probability-based feature set by using DT and BiLTCN. The generated feature set is more linearly separable and distinguishes the target classes with a higher margin.

**Fig 13:**
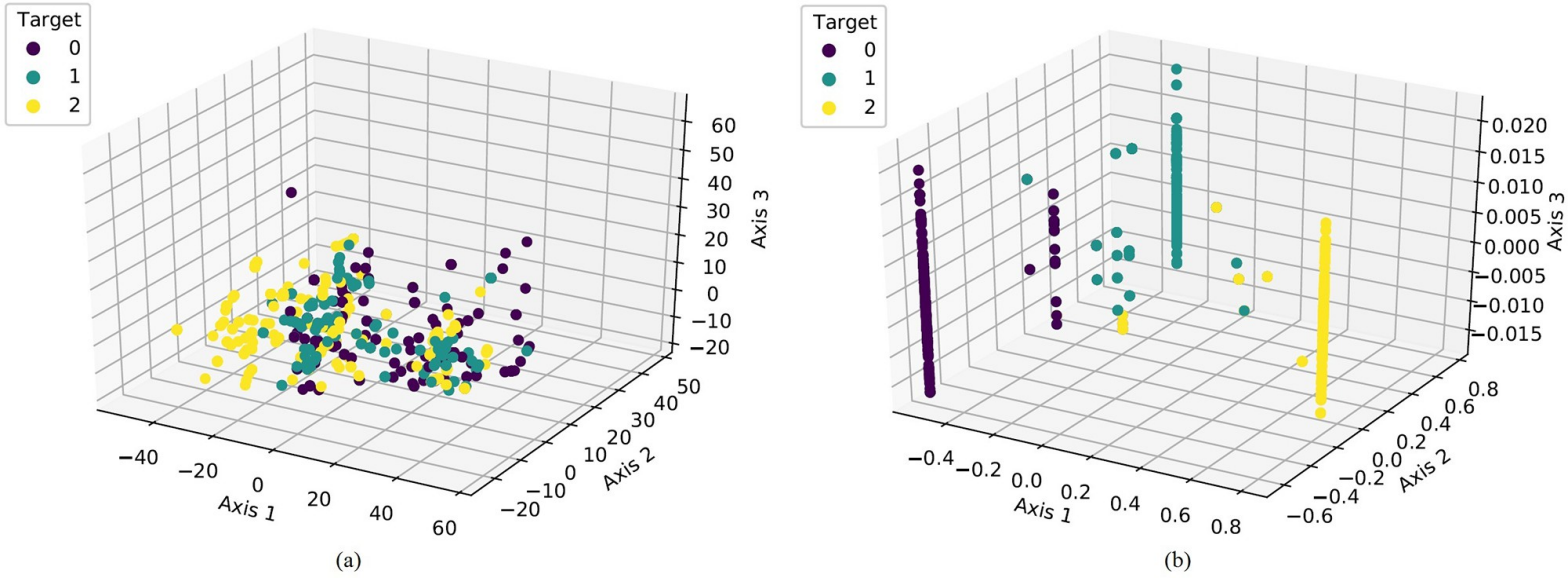
The feature space analysis is based on class distribution using the original features and class distribution using the proposed DTBiLTCN.

The experimental results demonstrate that by using the DTBiLTCN technique, the performance of the machine learning models is enhanced greatly. A visual representation of feature distribution is analyzed to verify the enhanced performance, as given in Fig 13. Fig 13a demonstrates that the dataset with the original feature is not linearly separable. However, by using the proposed feature engineering technique the feature space becomes more linearly separable as visualized in Fig 13b. This analysis validates the performance enhancement by using the proposed feature engineering technique.

We also determined the computational cost for the applied machine learning model with the original and probability-based feature set. The analysis demonstrates that the proposed approach is significant in terms of accuracy as well as efficiency. The computational cost for the proposed technique is significantly better than other applied techniques. The highest accuracy is achieved with the lowest computational time, as analyzed in Table 15.

**Table 15:** The computational cost analysis (time in seconds) of machine learning methods.

Technique	Original Features	DTBiLTCN Features
DTC	0.06	0.01
LR	0.05	0.02
ETC	0.51	0.40
KNN	0.01	0.01
RFC	0.65	0.63
**SVM**	**0.79**	**0.04**

### Performance comparison with existing approaches

The comparative analysis of the existing state-of-the-art approaches in the context of maternal health risk prediction is demonstrated in Table 16. For this purpose, recent research works are compared with the proposed approach. The selected works include both machine learning and deep learning models. Performance analysis corroborates the superior performance of machine learning models when used with the proposed DT-BiLTCN feature extraction approach.

**Table 16:** Performance analysis with existing approaches.

Reference	Year	Framework tool	Proposed technique	Accuracy%
[51]	2017	Python	TF-IDF + LSVM	92
[17]	2020	Python and WEKA	Decision tree learning	97
[52]	2020	Python and WEKA	Logistic model tree	90
[53]	2021	Python	Relief-F + CNN	88
[54]	2021	Python	Chi-Squared + BPM	96
[55]	2021	Python	TF-IDF,BoW + ETC	93
**Proposed**	**2022**	**Python**	**DT-BiLTCN + SVM**	**98**

### Discussions

This study proposes a novel deep neural network architecture that leverages DT, Bi-LSTM, and TCN networks to make an ensemble for feature engineering. Based on the individual superior performance of these models, they are combined for feature engineering to predict maternal health risk during pregnancy. Empirically, the performance of prediction probability is found superior to that of original features. So, the feature set comprises the prediction probabilities from these models. For class imbalance, SMOTE is utilized. Experiments are performed using imbalanced and balanced data, with original features from the data and the features extracted using the proposed approach. Extensive experiments and analyses show that the proposed approach is superior regarding maternal health risk prediction. SMOTE-balanced data tend to show better results for a few models while the performance of the proposed approach is substantially elevated. Experimental results using DTC, LR, ETC, KNN, RFC, and SVM with the proposed DT-BiLTCN feature engineering approach show that accuracy, precision, recall, and F1 scores of 0.98 can be obtained when SVM is used with the proposed approach. This performance is significantly better than using the original features from the dataset. In addition, k-fold cross-validation results validate this performance. Performance comparison with existing state-of-the-art approaches further corroborates these results.

## Conclusions and future work

Maternal health analysis and risk detection during pregnancy are very important to reduce the probability of health complications during pregnancy, childbirth, and postpartum periods. This study presents a novel approach for the automatic prediction of maternal health risks associated with pregnancy. A novel approach DT-BiLTCN is proposed for feature extraction which provides better features for machine learning models’ effective training. Of the employed models, SVM shows an excellent accuracy of 98% for low, medium, and high-risk categories of maternal health risks. When compared with the performance of DT and BiLTCN features, the proposed approach outperforms them regarding ROC and other metrics. Regarding, maternal health exploratory data analysis, the diastolic and systolic blood pressure, heart rate, and age of pregnant women are found to be the key health factors that can cause high-risk levels during pregnancy. We believe that the findings of this study are helpful for doctors, maternal health professionals, and the government to predict the risk factors associated with maternal mortality and can be used to alleviate such outcomes.

## Supporting information

S1 Dataset (ZIP)
